# Emergency Department Presentations for Low Back Pain by Remoteness and Socioeconomic Status in New South Wales: A Population‐Based Study

**DOI:** 10.1111/1742-6723.70127

**Published:** 2025-08-20

**Authors:** Jacqueline North, Zoe A. Michaleff, Michael Lee, Christopher Williams, Alexandre S. Stephens

**Affiliations:** ^1^ School of Health Sciences, Faculty of Medicine and Health The University of New South Wales Sydney New South Wales Australia; ^2^ Northern New South Wales Local Health District Lismore New South Wales Australia; ^3^ University Centre for Rural Health, School of Health Sciences, The University of Sydney Lismore New South Wales Australia; ^4^ Mid North Coast Local Health District Port Macquarie New South Wales Australia; ^5^ Sydney Medical School, Faculty of Medicine and Health The University of Sydney Sydney New South Wales Australia

**Keywords:** hospital emergency service, low back pain, rural health, socioeconomic factors

## Abstract

**Objective:**

To explore the patterns of emergency department (ED) presentations for low back pain (LBP) by remoteness and socioeconomic status (SES) in New South Wales (NSW).

**Methods:**

A retrospective, population‐based study of deidentified data was undertaken with data sourced from the NSW Emergency Department Data Collection (EDDC). The study population comprised NSW residents who presented to an NSW public hospital ED in 2013–2019 for LBP and were registered in the NSW EDDC. Total LBP ED presentations, quasi‐Poisson regression modelled annual changes in LBP ED presentations over 2013–2019, and age and sex‐standardised rates of ED presentations in 2019 were assessed.

**Results:**

Overall, between 2013 and 2019, LBP ED presentations increased in both metropolitan and rural NSW, with mean annual percentage increases of 3.5% (95% CI 2.9–4.0) and 4.7% (95% CI 3.6–5.9), respectively. Rates of LBP ED presentations increased with decreasing SES in both metropolitan and rural areas. Rates of LBP ED presentations were higher in rural areas compared to metropolitan NSW across all SES quintiles.

**Conclusions:**

Remoteness and SES were independently associated with rates of LBP ED presentations. Further research on the underlying causal mechanisms linking rates of LBP to each of these key sociodemographic factors is warranted. Elucidating these mechanisms would provide crucial information to inform strategies to reduce the rates and impacts of LBP presentations in EDs and help counteract social determinants of health.

## 1 Introduction

Low back pain (LBP) is a common problem associated with considerable healthcare burden and a large human cost through discomfort, physical disability, and lost productivity [1, 2, 3]. As an indicator of its prevalence, in 2020, 619 million individuals were estimated to be affected by LBP globally [4]. In terms of healthcare burden, LBP has been identified as the single leading cause of disability worldwide and, when grouped within the broader category of generalised back pain, it was the 6th most common reason for presenting to an emergency department (ED) in Australia in 2022–23 [5]. Its sizeable burden translates to considerable healthcare costs in Australia [6], a phenomenon observed in other countries [3].

Better understanding the preceding factors that lead to LBP and its outcomes following care would provide crucial information to inform preventative strategies and care options. Despite the strong evidence base that exists for treating and managing LBP [7, 8], there remains considerable variation in how patients are clinically managed [9]. There is also evidence linking LBP outcomes with social determinants of health [10, 11, 12], which captures how societal structures, such as social and economic conditions, act as preceding factors that heavily influence opportunities for education, employment, housing, lifestyle choices, and access to care, ultimately impacting health [10, 11, 13, 14]. In the case of socioeconomic status (SES), it is uncertain to what degree its impact is confounded by remoteness and what the independent effects of the factors are once adjusted for one another. Better understanding the independent effects of remoteness and SES on LBP ED presentations will help to define and identify the strategic approaches to prevention and management of the condition. The aim of this study was to explore patterns of ED presentations for LBP by remoteness and SES in NSW using age‐sex standardised rates of LBP ED presentations. Multivariable statistical models were also used to estimate the independent effects of remoteness and SES on rates of LBP ED presentations.

## 2 Methods

### 2.1 Setting

The study population included NSW residents that presented to an NSW public hospital ED in 2013–2019 for LBP (ICD‐10 code M54.5) and were registered in the NSW Emergency Department Data Collection (EDDC). The EDDC contains unit record, de‐identified clinical and sociodemographic information for each ED presentation and was established for the purposes of monitoring patient presentations to and the activity undertaken in EDs of NSW public hospitals. A total of 173 EDs from across the entire state, inclusive of all NSW Local Health Districts and Specialty Networks, contributed data to the study. For trend analysis, data were restricted to 141 EDs (36 from metropolitan NSW and 105 from rural NSW) that continuously reported to the EDDC between 2013 and 2019 (the study period).

### 2.2 Data

Study data were obtained from the EDDC as a component‐linked dataset of the Admitted Patient, Emergency Department and Deaths Register (APEDDR) and analysed longitudinally and cross‐sectionally. Planned presentations (1.74% of all ED presentations during the study period) were excluded from analyses. The EDDC was the source of age group (5‐year up to open‐ended group of 85 years or more), remoteness and sex. Remoteness of residence was classified as metropolitan or rural (comprising inner regional, outer regional, remote and very remote areas) in accordance with the Accessibility and Remoteness Index of Australia Plus (ARIA+) [15]. ARIA+ is the official classification of remoteness used by the Australian Bureau of Statistics (ABS) with values ranging from 0 (high accessibility) to 15 (high remoteness) [15]. SES was assigned to EDDC records by Statistical Area Level 1 (SA1) geography by special request to the NSW Ministry of Health. Therefore, SES assigned to records was based on location of residence, reflecting the average SES of the SA1 they reside in (generally between 200 to 800 persons) [16] as opposed to individual‐level SES. Quintiles of SES derived from the ABS Socioeconomic Indexes for Areas (SEIFA) Index of Relative Socioeconomic Disadvantage (IRSD) were used in analyses and range from Quintile 1, most disadvantaged, to Quintile 5, most advantaged [17]. The IRSD is a validated composite measure of disadvantage and consists of variables on housing, income, education, employment and occupation [17]. A small fraction of records (< 1%) were missing geography information and could not be assigned a valid SA1‐level SES measure or remoteness and accounts for the small discrepancies between total LBP ED presentations in NSW and those summed across metropolitan and rural areas (e.g., as per Table 1).

**TABLE 1:** Low back pain ED presentations, and their breakdown by SES (% in parentheses), in 2013 and 2019, and total changes and annual mean and percentage (%) mean changes between 2013 and 2019 for all NSW and metropolitan and rural NSW.

	NSW					Metropolitan					Rural				
	Years			Annual change^a^		Years			Annual change^a^		Years			Annual change^a^	
	2013^b^	2019^b^	Change	Mean	%	2013^b^	2019^b^	Change	Mean	%	2013^b^	2019^b^	Change	Mean	%
All	15,222 (100.0)	18,965 (100.0)	3743	578	4.0 [3.3–4.7]	8680 (100.0)	10,515 (100.0)	1835	287	3.5 [2.9–4.0]	6515 (100.0)	8392 (100.0)	1877	287	4.7 [3.6–5.9]
SES quintile															
1	5165 (33.9)	6406 (33.8)	1241	193	4.0 [3.0–5.0]	2361 (27.2)	2944 (28.0)	583	92	4.2 [3.2–5.2]	2805 (43.0)	3462 (41.2)	657	100	3.8 [2.5–5.1]
2	3698 (24.3)	4747 (25.0)	1049	149	4.2 [3.4–5.0]	1978 (22.8)	2445 (23.2)	467	64	3.3 [2.6–4.1]	1720 (26.4)	2302 (27.4)	582	84	5.2 [3.9–6.5]
3	2635 (17.3)	3192 (16.8)	557	92	3.7 [2.8–4.5]	1567 (18.1)	1855 (17.6)	288	50	3.3 [2.2–4.5]	1068 (16.4)	1337 (15.9)	269	41	4.2 [2.9–5.4]
4	1994 (13.1)	2506 (13.2)	512	83	4.4 [3.2–5.7]	1321 (15.2)	1585 (15.1)	264	40	3.1 [2.0–4.3]	673 (10.3)	921 (11.0)	248	40	7.0 [4.8–9.2]
5	1624 (10.7)	1981 (10.4)	357	58	3.7 [3.2–4.3]	1408 (16.2)	1622 (15.4)	214	36	2.7 [2.3–3.0]	216 (3.3)	359 (4.3)	143	19	9.7 [7.2–12.3]

^a^ Mean and percentage (%) mean changes were based on quasi‐Poisson regression models (with dispersion parameter) fit to ED presentation data between 2013 and 2019 (inclusive of all calendar years). For annual mean percentage (%) changes, the 95% confidence interval of the estimates are displayed in square brackets.

^b^ The percentage distributions of total ED presentations by SES quintiles are displayed in parentheses.

### 2.3 Mapping of Systematised Nomenclature of Medicine–Clinical Terms (SNOMED‐CT) to International Classification of Diseases 10th Revision (ICD‐10)

Five (5) coding classifications were used to code the principal (and only) diagnosis field (the diagnosis or condition established after assessment to be responsible for the person presenting to the Emergency Department) in the ED data analysed in the study. These were SNOMED‐CT, ICD10AM, ICD9CM, ICD10V8, and ICD10V9. For each individual calendar year over the study period, SNOMED‐CT and ICD10AM were used to code 96% or more of all ED presentations, with SNOMED‐CT coding between 75% and 81% of diagnoses. To identify SNOMED‐CT codes that corresponded to LBP presentations, codes were mapped to ICD‐10 using the Unified Medical Language System (UMLS) SNOMED CT to ICD‐10‐CM Map [18]. The mapping identified 13 SNOMED‐CT codes that corresponded to ICD‐10 code M54.5 (LBP). The codes, their names, and frequencies (i.e., the number of records they were used to code the ED diagnosis) across the study period (summed across all calendar years) are available for viewing in Table S1. Ethical approval was obtained from a certified Human Research Ethics Committee (2020/ETH00054/2020.09) prior to the commencement of this study.

### 2.4 Analysis

Data were analysed and reported by absolute numbers or derivatives of ED presentations (e.g., differences, mean changes and % mean changes) with 95% confidence intervals (95% CIs). For the analysis of trends over time, while all data between 2013 and 2019 were used, data for 2013 and 2019 (totals and proportional distribution across SES quintiles) are reported in Table 1 to act as frames of reference and aid in interpretation. Mean and percentage (%) mean changes in ED presentations between 2013 and 2019 were based on quasi‐Poisson regression models (with dispersion parameter) fit to ED presentation data (inclusive of all data between 2013 and 2019). A standard log link function was applied, and presentations were modelled using calendar year as a continuous variable that was assumed to be linearly related to the outcome. As only SNOMED‐CT and ICD10AM were used to flag LBP ED presentations, numbers of LBP presentations were likely slightly underestimated over the study period. To assess the potential impact of this, a sensitivity analysis on an adjusted dataset derived by multiplying the total number of LBP presentations by SES quintile and remoteness strata groups by the factor by which ED presentations were underestimated by year based on the proportion of all presentations not coded as SNOMED‐CT or ICD10AM was undertaken. This analysis assumed that the proportion of records not coded as SNOMED‐CT or ICD10AM by year was consistent across strata groups defined by the main study factors (i.e., SES and remoteness). The findings are presented in Table S2. For the analysis of age‐standardised rates and statistical modelling of remoteness and SES effects, all LBP EDDC data for 2019 were used (where reporting to the EDDC was near complete and the proportion of ED records whose diagnoses were not coded using ICD‐10 or SNOMED‐CT was < 1%). Directly age‐standardised rates and age‐specific rates of LBP ED presentations per 100,000, with Poisson‐based variances adjusting for multiple events [19], were calculated using the Australian residential population as of 30 June 2011 as the standard population. Negative binomial regression models, which cater for increased variance associated with multiple events [20], were applied to estimate the effects of remoteness and SES on rates of LBP presentations. A standard log link function was applied, and presentations were modelled using fixed terms for age group, sex, SES and remoteness. As the intention of the analysis was to estimate the (average) effects of the fixed terms on rates of LBP ED presentations, data were aggregated across strata groups defined by the combinations of the fixed terms prior to analysis. Log estimated residential populations (ERPs) for the strata groups obtained through the aggregation of SA1 ERPs, to which single SA1‐level measures of SES and remoteness were assigned (enabling data to be aggregated across strata groups defined by age group, sex, remoteness and SES quintile), were included in models as offset variables. Rate ratios (RRs) were calculated and denote the fold change in rates of LBP ED presentations when a particular study factor is present and can be interpreted as the multiplication factor by which rates are changed when the study factor is present compared to not. Two‐way interactions between sex and age group, and remoteness and SES quintiles were included in final statistical models. Estimated effects are presented as RRs with 95% CIs. Model fits were assessed using deviance statistics and the Akaike information criterion (AIC). Data manipulations were performed in SAS Enterprise Guide version 8.3 (SAS Institute, Cary NC) and statistical models and figures were generated in R version 4.3.0 with RStudio 2024.04.2+764 ‘Chocolate Cosmos’.

## 3 Results

### 3.1 Study Sample

In 2013, the ERP in NSW was 7,404,032 and there was a total of 15,222 ED presentations for LBP, with 8680 in metropolitan NSW and 6515 in rural NSW. In 2019, the NSW ERP was 8,046,748 (8% increase compared to 2013) and LBP ED presentations increased to a total of 18,965, with 10,515 and 8392 in metropolitan and rural NSW, respectively, representing absolute increases of 1838 (21%) and 1877 (29%) relative to 2013, respectively (Table 1), and mean annual percentage changes of 3.5% (95% CI 2.9–4.0) and 4.7% (95% CI 3.6–5.9), respectively (Table 1). When disaggregated by SES quintiles, most LBP presentations in rural NSW were from people residing in the most disadvantaged SA1s (e.g., in 2019, 5764 [68.6%] of presentations were from people that resided in SA1s classified as SES quintiles 1 and 2). Although more evenly distributed across SES quintiles, in metropolitan NSW, people residing in SA1s classified as SES quintiles 1 and 2 accounted for 51.2% of all LBP ED presentations in 2019. For mean annual percentage changes, gradients along SES quintiles were observed in both metropolitan and rural NSW, with changes decreasing with increasing SES in metropolitan NSW and, conversely, changes increasing with increasing SES quintile in rural NSW (Table 1).

### 3.2 Age‐ and Sex‐Standardised and Age‐Specific Rates of ED Presentations

**FIGURE 1:**
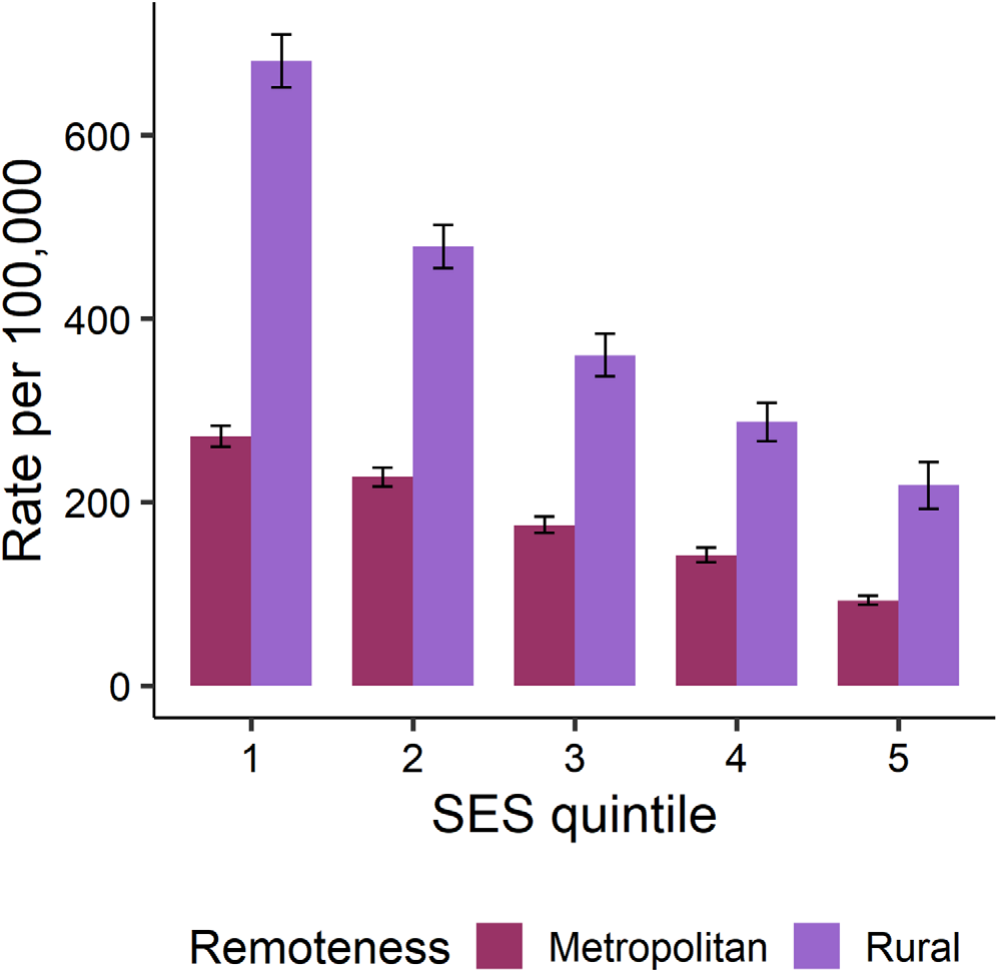
Age‐ and sex‐standardised rates of low back pain ED presentations by SES quintiles and remoteness, NSW, 2019. SES, socioeconomic status.

Figure 1 displays age‐ and sex‐standardised rates of LBP ED presentations by SES quintile and remoteness in NSW in 2019. Rates of LBP ED presentations were higher in rural NSW at each SES quintile, denoting the remoteness effects. For SES, rates were highest in the most disadvantaged quintiles (SES 1) and progressively decreased with increasing SES, and therefore, displayed negative gradients with increasing SES in both rural and metropolitan NSW. To provide a quantitative view of these findings, a negative binomial regression model was fit to the data and the arising RRs are reported in Table 2. In SES 1, the rates of LBP ED presentations were 2.30 times greater in rural areas compared to metropolitan areas. Rural increases in rates of LBP ED presentations were observed in other SES quintiles, but to a lesser degree than SES 1, with the second most pronounced effect in SES 5 (Table 2). For SES effects, as noted above, negative gradients were observed, manifesting as rates as high as 2.75 (95% CI 2.46–3.08) and 2.88 (95% CI 2.49–3.32) times greater than those observed in SES 5 (the reference category) in SES 1 in metropolitan and rural NSW, respectively (Table 2).

**TABLE 2:** Estimated effects (rate ratios) of remoteness and socioeconomic status (SES) on rates of low back pain ED presentations conditional on each other with 95% confidence intervals (in square brackets) NSW, 2019.

Effect	All presentations
Remoteness by SES quintile (relative to major city areas)	
Q1	2.30 [2.08–2.56]
Q2	1.93 [1.73–2.15]
Q3	1.93 [1.72–2.17]
Q4	1.91 [1.69–2.16]
Q5	2.21 [1.90–2.56]
SES in metropolitan areas (relative to Q5)	
Q1	2.75 [2.46–3.08]
Q2	2.37 [2.12–2.66]
Q3	1.85 [1.65–2.08]
Q4	1.53 [1.36–1.72]
SES in regional and remote areas (relative to Q5)	
Q1	2.88 [2.49–3.32]
Q2	2.07 [1.79–2.40]
Q3	1.62 [1.39–1.88]
Q4	1.32 [1.13–1.55]

**FIGURE 2:**
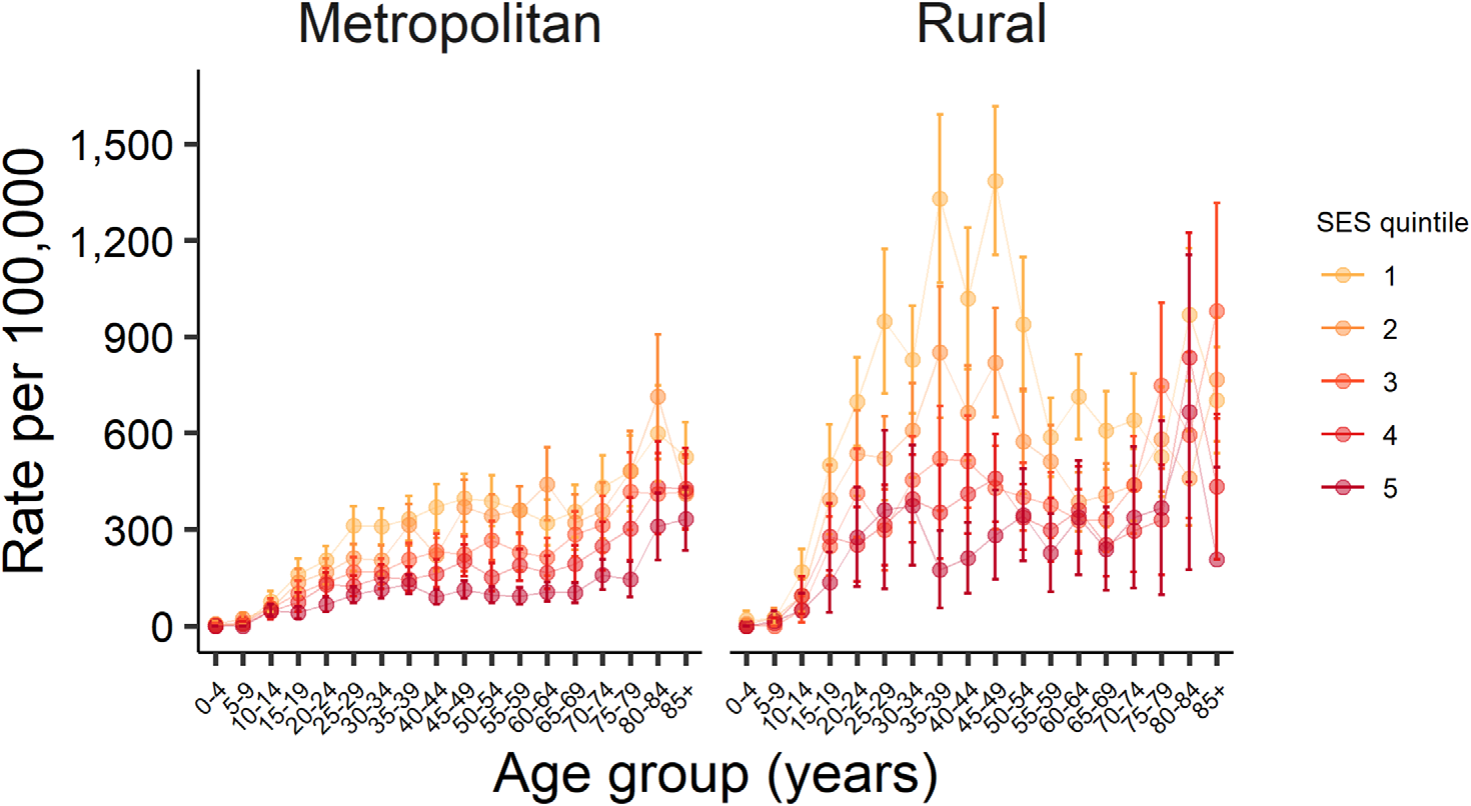
Age‐specific rates of low back pain ED presentations by SES quintiles and remoteness, females, NSW, 2019.

**FIGURE 3:**
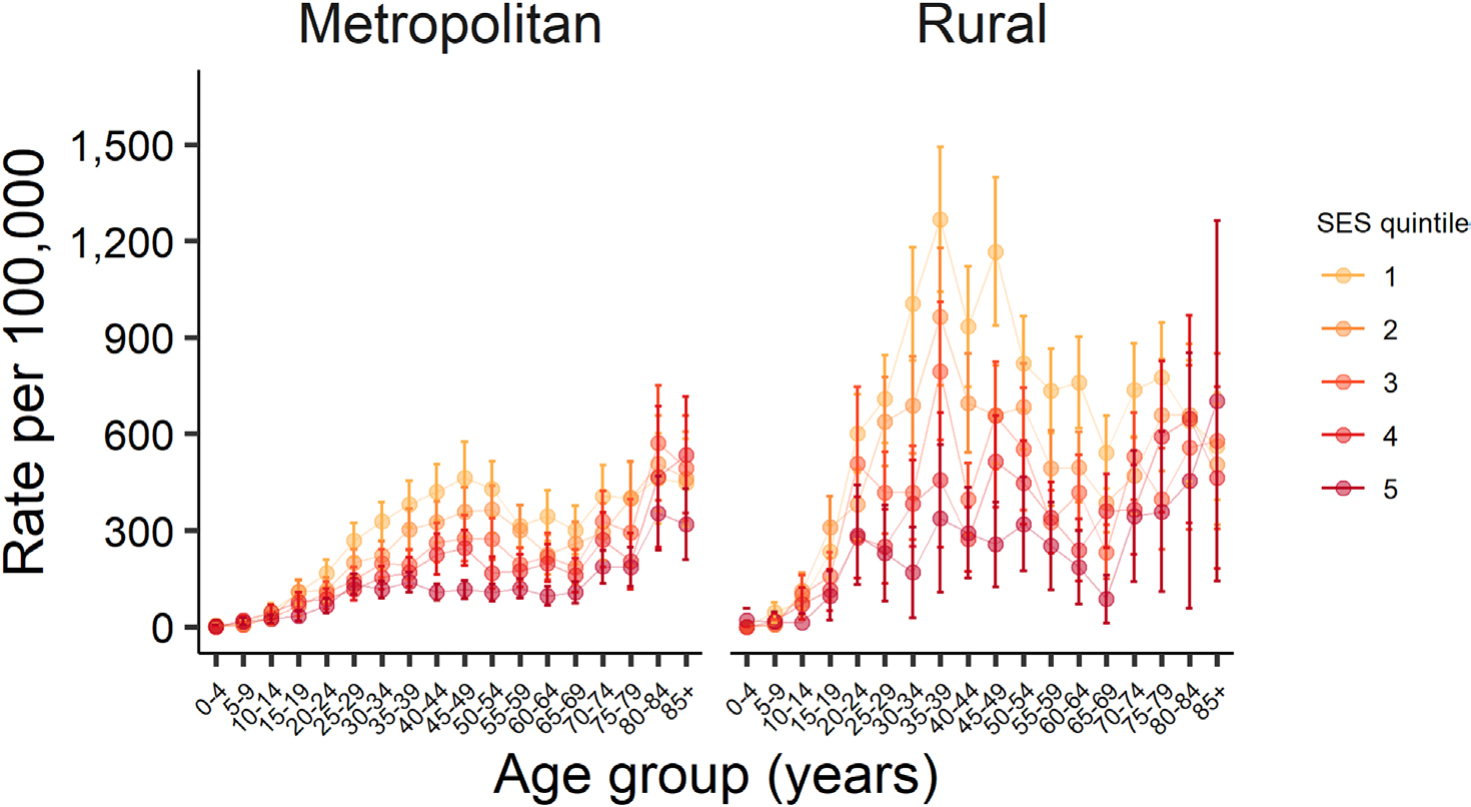
Age‐specific rates of low back pain ED presentations by SES quintiles and remoteness, males, NSW, 2019.

The analysis of age‐specific rates showed that LBP ED presentations varied by age group. SES effects also varied by age group for both males and females and were most pronounced in working age groups (~20–65 years of age) (Figures 2 and 3). Higher rates of LBP presentations were observed in both males and females in rural compared to metropolitan NSW and varied by age group (Figures 2 and 3).

## 4 Discussion

This study investigated LBP presentations to NSW EDs by remoteness and SES. Findings showed that ED presentations for LBP increased from 2013 to 2019 in both metropolitan and rural NSW, aligning with the general increase in demand for emergency services across NSW and Australia more broadly [21]. There were also dominant effects for remoteness and SES in determining rates of LBP presentations, both in an absolute and relative sense. Collectively, the study findings demonstrate key roles for remoteness and SES in determining rates of ED presentations for LBP, adding to the growing body of evidence on social determinants of health [10, 11].

Rates of LBP ED presentations were higher in rural areas compared to metropolitan NSW across all SES quintiles. One potential reason for these higher rates is the limited access to primary care in rural areas [22, 23], which may also broadly explain, at least to some degree, the generally higher rates of all low acuity ED activity in rural areas [21]. The primary care health workforce, including general practitioners and physiotherapists, is unevenly distributed geographically across Australia, with rates per capita generally lower in rural areas [22, 23]. Primary healthcare professionals are well placed to manage LBP in the community [7, 8] often acting as the initial contact point for health management. Challenges in accessing them by patients experiencing LBP may force them to present to EDs to receive care. Taking steps to better balance the healthcare workforce distribution may alleviate the pressure on EDs, but requires the identified barriers to growing the rural workforce to be addressed, including a lack of career opportunities and distance from family and other social networks [22, 23].

Known primary healthcare workforce shortages are an ongoing concern in Australia, which the Australian Government is attempting to address. The Australian Government's Stronger Rural Health Strategy is aiming to attract and retain healthcare professionals in rural areas and improve access to healthcare services [24]. Australia's Primary Health Care 10 Year Plan 2022–2032 is aiming to strengthen primary healthcare services, with a focus on preventive care and early intervention [25]. Strategies such as higher remuneration and financial support for healthcare workers, as identified in the 2023 Australian Physiotherapy Association (APA) Workforce Census, may also assist healthcare workers to relocate to rural or remote areas [22].

Increasing the scope of practice of certain healthcare professionals, such as allied health and pharmacists, and exploring alternative models of healthcare delivery may also improve the capacity of healthcare professionals to address low acuity conditions such as LBP that may otherwise present to ED [26, 27].

Rates of LBP ED presentations increased with decreasing SES in both metropolitan and rural areas. These findings add to the growing body of evidence linking key health outcomes to social determinants [10, 11, 28, 29]. It is now well recognised and accepted that the way our societies are structured and that one's position in society and their opportunities for education and employment have a direct bearing on access to housing and clean and safe living conditions [28, 29, 30]. In turn, these influence health risk behaviours and lifestyle choices, which ultimately impact health [28, 29, 30]. Addressing health inequities stemming from social determinants remains an ongoing challenge for governments and health organisations despite the existence of established frameworks (Fair Society, Healthy Lives [31]; Global Plan of Action on Social Determinants of Health [13]) to address them. In recognition of these ongoing challenges and persisting health inequalities, the World Health Organization Seventy‐fourth World Health Assembly, through resolution 74.16, requested the release of a ‘World Report on Social Determinants of Health Equity’, capturing the recommendations for ongoing action of member organisations to strengthen efforts to address social determinants of health and associated health inequities [32]. A focus on addressing social determinants of health should remain a top priority for governments. A key consideration would be to focus on efforts where inequalities are most pronounced, which, based on the study findings, appears to be in the working age groups for LBP.

### 4.1 Strengths and Limitations

The study had several strengths and limitations. The main strength was the use of SA1‐SEIFA data to assign SES, which improved the granularity of geographical detail in the data analysis. SA1s are the smallest area for which the indexes are available from the ABS and represent approximately 400 people on average; whereas Statistical Area Level 2 (SA2s) are medium‐sized geographic areas that represent approximately 10,000 people on average. Analysis of SA1s enabled a more detailed understanding of socioeconomic trends within localised regions of NSW [33]. However, individual levels of disadvantage cannot be implied as SEIFA measures relative advantage and disadvantage at a geographic area level [33].

A limitation of this study was that it only included presentations that were coded as LBP (ICD‐10 M54.5 or SNOMED‐CT codes that mapped to ICD‐10 M54.5), which explains the differences in LBP prevalence in ED presentations to a similar study that analysed NSW ED records [12]. This code was deliberately used to focus on non‐specific LBP, which represents the most common LBP presentation to NSW EDs, a condition that should be preferentially managed in primary care settings. Excluding alternative ICD codes related to LBP presentations would have reduced the sample population in this study and, considering this, limits the generalisability of the study findings to non‐specific LBP. The reliance on accurate and valid data entry during presentation to ED is another potential limitation of this study. Planned LBP presentations were excluded from data analysis; however, these accounted for a small percentage of LBP ED presentations during the study period (1.74%). Private hospital ED data was not available for analysis, which may have caused an underestimation in LBP presentations, particularly in higher SES quintiles that are more likely to access them and in metropolitan areas where private EDs are more available. Only two broad areas of geography, rural and metropolitan NSW, were explored. Further research is needed to understand SES and remoteness effects at more subtle geographical levels.

### 4.2 Conclusions

Negative gradients of LBP ED presentations across the SES spectrum were observed in both metropolitan and rural areas. At each SES quintile studied, rates of LBP ED presentations were higher in rural areas. Collectively, the study findings demonstrate important and independent roles for remoteness and SES in explaining rates of LBP ED presentations. The findings add to the growing body of evidence linking social determinants to a range of health outcomes and highlight the need for governments to act. Further research to better understand the underlying causal pathways for each factor is warranted to establish preventative strategies and care options.

## Conflicts of Interest

The authors declare no conflicts of interest.

## Supporting information


**Data S1:** Supporting Information.

## Data Availability

The data that support the findings of this study are available on request from the corresponding author. The data are not publicly available due to privacy or ethical restrictions.
