# Supplementary material for: Emergency Department Presentations for Low Back Pain by Remoteness and Socioeconomic Status in New South Wales: A Population‐Based Study
Source: Emerg Med Australas. 2025 Aug 20;37(4):e70127. doi: 10.1111/1742-6723.70127 (PMC12365860; doi:10.1111/1742-6723.70127)
Supplement: Supplementary file 1 — Data S1: Supporting Information. [file EMM-37-0-s001.docx]

**Emergency Department Presentations for Low Back Pain by Remoteness and Socioeconomic Status in New South Wales: A Population-Based Study**

**Supplementary material**

**Supplementary Table 1:** SNOMED-CT codes and names that map to ICD10 M54.5 low back pain and their frequencies (i.e. how frequently they were used to code diagnoses in emergency department records) across the study period (2013-2019).

| **SNOMED-CT code** | **Name** | **Frequency*** |
| --- | --- | --- |
| 279039007 | Low back pain | 75120 |
| 271857006 | Loin pain | 1817 |
| 61486003 | Sacral back pain | 1778 |
| 278862001 | Acute low back pain | 1755 |
| 278860009 | Chronic low back pain | 546 |
| 279040009 | Mechanical low back pain | 401 |
| 161894002 | C/O - low back pain | 139 |
| 272047006 | C/O - loin pain | 11 |
| 298674008 | Lumbar spine painful on movement | 6 |
| 267982002 | Pain in lumbar spine | 4 |
| 279063004 | Lumbar facet joint pain | 2 |
| 427475007 | Pain radiating to lumbar region of back | 2 |
| 298673002 | Lumbar spine - tender | 1 |

*Between 2013 and 2019, 36,558 records had a diagnosis of M54.5 low back pain (coded using ICD10AM).

**Sensitivity analysis of an adjusted dataset derived by multiplying the total number of LBP presentations by SES quintile and remoteness strata groups by the factor by which ED presentations were underestimated by year based on the proportion of all presentations not coded as SNOMED-CT or ICD10AM across 2013 to 2019.**

**Supplementary Table 2:** Low back pain ED presentations, and their breakdown by SES (% in parentheses), in 2013 and 2019, and total changes and annual mean and percentage (%) mean changes between 2013 and 2019 for all NSW and metropolitan and rural NSW based on the analysis of an adjusted dataset.

|  | NSW | | | | |  | Metropolitan | | | | |  | Rural | | | | |
| --- | --- | --- | --- | --- | --- | --- | --- | --- | --- | --- | --- | --- | --- | --- | --- | --- | --- |
|  | Years | |  | Annual change**^†^** | |  | Years | |  | Annual change**^†^** | |  | Years | |  | Annual change**^†^** | |
|  | 2013**^‡^** | 2019**^‡^** | Change | Mean | % |  | 2013**^‡^** | 2019**^‡^** | Change | Mean | % |  | 2013**^‡^** | 2019**^‡^** | Change | Mean | % |
| All | 15869 (100.0) | 19145 (100.0) | 3276 | 510 | 3.4 [2.6-4.2] |  | 9049 (100.0) | 10615 (100.0) | 1566 | 245 | 2.8 [2.2-3.5] |  | 6792 (100.0) | 8472 (100.0) | 1680 | 259 | 4.1 [2.8-5.3] |
| SES quintile | |  |  |  |  |  |  |  |  |  |  |  |  |  |  |  |  |
| 1 | 5385 (33.9) | 6467 (33.8) | 1082 | 169 | 3.3 [2.3-4.4] |  | 2461 (27.2) | 2972 (28.0) | 511 | 82 | 3.6 [2.5-4.6] |  | 2924 (43.0) | 3495 (41.2) | 571 | 87 | 3.1 [1.8-4.5] |
| 2 | 3855 (24.3) | 4792 (25.0) | 937 | 132 | 3.6 [2.6-4.6] |  | 2062 (22.8) | 2468 (23.2) | 406 | 54 | 2.7 [1.7-3.7] |  | 1793 (26.4) | 2324 (27.4) | 531 | 77 | 4.6 [3.2-5.9] |
| 3 | 2747 (17.3) | 3222 (16.8) | 475 | 79 | 3.0 [2.1-4.0] |  | 1634 (18.1) | 1873 (17.6) | 239 | 43 | 2.7 [1.6-3.8] |  | 1113 (16.4) | 1350 (15.9) | 237 | 37 | 3.5 [2.1-4.9] |
| 4 | 2079 (13.1) | 2530 (13.2) | 451 | 74 | 3.8 [2.4-5.2] |  | 1377 (15.2) | 1600 (15.1) | 223 | 34 | 2.5 [1.2-3.8] |  | 702 (10.3) | 930 (11.0) | 228 | 38 | 6.3 [4.1-8.6] |
| 5 | 1693 (10.7) | 2000 (10.4) | 307 | 50 | 3.1 [2.5-3.7] |  | 1468 (16.2) | 1637 (15.4) | 169 | 29 | 2.0 [1.7-2.3] |  | 225 (3.3) | 362 (4.3) | 137 | 18 | 9.1 [6.5-11.7] |

^†^Mean and percentage (%) mean changes were based on quasi-Poisson regression models (with dispersion parameter) fit to ED presentation data between 2013 and 2019 (inclusive of all calendar years). For annual mean percentage (%) changes, the 95% confidence interval of the estimates are displayed in square brackets. ^‡^The percentage distributions of total ED presentations by SES quintiles are displayed in parentheses.
